# The Dual Importance of Virtual Reality Usability in Rehabilitation: A Focus on Therapists and Patients

**DOI:** 10.7759/cureus.56724

**Published:** 2024-03-22

**Authors:** Waqar M Naqvi, Ifat Naqvi, Gaurav V Mishra, Vishnu Vardhan

**Affiliations:** 1 Interdisciplinary Sciences, Datta Meghe Institute of Higher Education and Research, Wardha, IND; 2 Physiotherapy, College of Health Sciences, Gulf Medical University, Ajman, ARE; 3 Physiotherapy, Ravi Nair Physiotherapy College, Datta Meghe Institute of Higher Education and Research, Nagpur, IND; 4 Radiodiagnosis, Datta Meghe Institute of Higher Education and Research, Wardha, IND; 5 Physiotherapy, Ravi Nair Physiotherapy College, Datta Meghe Institute of Higher Education and Research, Wardha, IND

**Keywords:** augmented reality (ar), usability, rehabilitation, user experience, virtual reality

## Abstract

Virtual reality (VR) has advanced in medical education and rehabilitation from basic graphical applications due to its ability to generate a virtual three-dimensional (3D) environment. This environment is mostly used to practice professional skills, plan surgery procedures, simulate surgeries, display 3D anatomy, and rehabilitate various disorders. VR has transformed the field of rehabilitation therapy by providing immersive and engaging experiences that go beyond traditional bounds, significantly improving patient care and therapeutic results. Considering the direct impact of VR on the efficacy of the treatment for both therapists and patients, its dual significance for usability and user experience cannot be overstated. The purpose of this article is to determine the synergistic association between VR accessibility and the rehabilitation process, highlighting the significance of VR technology in designing the future of rehabilitation therapy and demonstrating how advancing VR technology can improve therapeutic outcomes despite overcoming obstacles encountered during VR usage. In conclusion, VR offers a personalized, efficient, interesting, and engaging rehabilitative environment for patients, while also assisting therapists in cultivating empathy and efficiency and encouraging innovative approaches in treatment procedures.

## Introduction and background

Virtual reality (VR) has significantly evolved from basic graphical to advanced automotive, architecture, and medical applications. It has also been used in education, engineering, and production. These advances in VR have been made possible by the technology's potential to create a standardized, reproducible, and controllable environment [1,2]. In the modern medical education and treatment landscape, VR has gained recognition for its ability to create a simulated three-dimensional (3D) environment. This environment is mainly used for displaying 3D anatomy, practicing medical skills, surgical planning, simulating surgery, treating phobias, anxiety disorders, post-traumatic stress disorder, disabilities, and rehabilitation [2,3].

VR has revolutionized the field of rehabilitation therapy by providing immersive and interactive experiences that exceed conventional boundaries and greatly enhance patient outcomes and care. As VR technology becomes more widely used, its applications go beyond patient engagement and become essential tools for therapists to offer effective personalized therapy [4,5]. A multitude of studies and controlled trials have demonstrated the efficacy of VR in achieving therapeutic goals. These include the management of pain, developmental delay, Parkinson's disease, telerehabilitation and neurorehabilitation for stroke recovery, phobias, anxiety, and balance disorders highlighting the potential benefits of VR in medical care [6-8]. 

Since VR directly influences the effectiveness of therapy for both therapists and patients, its dual significance for usability and user experience cannot be overemphasized. Mastering VR technology for therapists is not merely about understanding a new tool; it’s about inculcating its potential to revolutionize rehabilitation procedures. This encompasses organizing sessions more efficiently, customizing exercises to meet individual patient needs, and gaining deeper insights into the patient’s experience of therapy. Simultaneously, patients gain from VR’s ability to design adaptable and realistic environments that fulfill their specific therapy expectations [4,5]. This article determines the mutually beneficial relationship between VR usability and the rehabilitation process, highlighting the role of VR technology in reshaping rehabilitation therapy in the future and illuminating how optimizing VR technology can enhance therapeutic outcomes.

## Review

The rise of VR in rehabilitation

VR and its application in rehabilitation is a recent and rapidly evolving interdisciplinary field where clinical science, basic science, and industry collaborate inter-professionally to boost scientific discovery and technological advancement leading to clinical implications [9,10]. In reality, execution is often so rapid that the provision of research and development priorities by funding agencies and the potential of researchers to demonstrate the efficacy of interventions tend to be reactive rather than proactive. Over the past 15 years, there has been a rapid increment in the sheer number and diversity of VR rehabilitation applications, which suggests that the study in this domain may be indicative of the emergence of a new scientific field that is primarily focused on the utility of VR technology to specific disabilities or impairments [9,11,12]. The field is progressing from being mainly concerned with developing new technologies to concentrating on how the technology can enhance rehabilitation objectives and principles [9]. Simulation technology and commercial gaming have been the primary forces behind these developments, with a growing number and variety of applications generated at the request of medical professionals and backed by multiple “start-ups” [9]. Traditionally, VR-based rehabilitation has been viewed as a dome requiring collaboration between clinical and behavioral scientists, engineers, and computer scientists [9]. 

VR provides incredibly immersive experiences that greatly improve treatment accomplishment and patient engagement. Studies have indicated that the immersive experience provided by VR enhances the motivation of the patient by diversifying rehabilitation tasks, providing multi-sensory feedback, and facilitating task-specific training [4,5]. VR serves a wide range of applications in rehabilitation for various disorders, including pain management, psychological treatment, and motor rehabilitation. Additionally, it elevates the standard of rehabilitation and opens up new avenues for patient-centered therapy, paving access to innovative approaches of therapy that are customized to the needs and progress of each patient individually [13,14]. Hence, the impact of VR-based rehabilitation would be significantly strengthened by the development of a strong research community to ensure valid and reliable results from the usage of VR.

Usability for therapists: enhancing efficiency and effectiveness

A crucial component of rehabilitation therapy that holds significant importance, especially for therapists in the modern world, is the usability of VR technology. A VR system that is intuitive and easy for users to navigate simplifies the process of therapy and also improves the effectiveness of treatment delivery. The easy process of setting up and using VR equipment is crucial for therapists since it reduces time spent on technicalities and enhances patient care and interaction. Research findings suggest that therapists who possess expertise in the utilization of VR technology have the capacity to customize rehabilitation programs more efficiently, thereby resulting in enhanced patient outcomes [4,5]. The effectiveness of therapy is increased when therapists utilize VR to construct real simulations that imitate everyday challenges, thus facilitating the development of practical skills and their transfer to real-life scenarios [9].

The commencement of VR-based rehabilitation offers the therapist the ability to more precisely regulate what is demonstrated to the patient, exposing patients to situations and objects that would be difficult to access or impractical, enhancing the patient’s ability to control the stimuli, decreasing embarrassment that patients may have after receiving therapies given in public, drawing more patients for VR-based rehabilitation, and improving treatment effectiveness in comparison to conventional forms of therapy. Additionally, it enables the therapists to reduce therapy sessions and increase treatment confidentiality that may be performed in public [15]. Furthermore, it assists therapists in managing smaller patient populations, monitoring and quantitative evaluation of patients, and optimizing therapy, all of which contribute to the successful completion of the intervention programs [16]. Moreover, therapists' preference for a wider range of VR games to prevent patients from losing interest during sessions and the incorporation of more gaming activities tailored to certain therapy procedures can maximize the effectiveness of rehabilitation [16].

VR-based rehabilitation by therapists is explained by eight attributes, namely learnability (easy to learn the system), appropriateness or usefulness (suitability of the product or system according to the patient’s need), effectiveness (accuracy and completeness by patients in achieving specified goals), efficiency (efficient system use for high-level productivity), memorability (easy to remember system functions), patient error protection and recovery (degree of protection against errors by the system), satisfaction (the system should be pleasant and acceptable), and operability (ability to control and operate the system software) [16,17]. 

Analyzing all the factors of VR-based rehabilitation provides important information regarding the usefulness and acceptability of the devices, consequently helping in understanding how to enhance the motivation of the patients during therapy sessions. Previous research has highlighted the critical impact that motivation plays in a rehabilitation program by helping the patient feel pleased and competent [18]. Furthermore, it was discovered that higher therapeutic compliance was related to the satisfaction of the patients with the therapeutic treatment [19]. The way patients respond to the virtual world may be greatly influenced by the selection of accurate and appropriate hardware and software by the therapists because of the unique features of the device. Moreover, therapists can concentrate on providing the highest level of physical assistance when necessary without diverting away from the difficulty of the task due to the delivery of the automated stimulus provided by the virtual settings [13]. Hence, based on the advancement in technology, VR is becoming a more valuable tool for therapists.

Customization: tailoring therapy to individual needs 

The key component for the effectiveness of VR-based rehabilitation therapy is customization according to the individual patient’s need, setting it apart from conventional rehabilitation methods. Since each patient is unique and has different experiences, strengths, and challenges, personalization is essential in addressing the specific challenges that each person faces during their recovery process. For example, VR can be designed to accommodate different levels of motor skills, psychological readiness, and cognitive abilities, offering a customized approach that acknowledges and reacts to the rehabilitation stage of each patient [20,21]. Moreover, the potential of the therapists to personalize VR experiences is essential for fulfilling the various demands of their patients. This consists of altering difficulty levels, virtual environments, and feedback mechanisms according to the individual recovery goals. Customization gives patients a sense of accomplishment and control, which benefits their psychological health in addition to their physical rehabilitation [22].

Customized VR-based rehabilitation has a significant impact, especially in neurological conditions. VR environments that imitate real-life tasks and circumstances are extremely beneficial to patients suffering from diseases such as stroke or traumatic brain injuries. This aids in the development of neuroplasticity and practical abilities [5,21] and facilitates an overall healing process by expediting both physical and cognitive rehabilitation. Additionally, VR's adaptability in rehabilitation is demonstrated by its use in pain management and psychological treatments by generating environments that are pleasant and distraction-based, which can enhance the whole therapeutic experience [4,22].

Customized VR systems for rehabilitation operate on computer-simulated 3D settings that enable the user to explore and engage through the combination of auditory, visual, and haptic feedback [23]. Accordingly, immersion, presence, and interaction are the three primary characteristics of VR [16]. Immersion is characterized as the extent to which VR can offer multisensory inputs that come from the virtual environment and a high degree of correspondence between the actions of the users and the cues produced by the system. Correspondingly, the patient’s experience, sensation of presence, and perception, are all influenced by the virtual environment that facilitates interaction and immersion [24]. VR-based rehabilitation can be customized into non-immersive comprising of tablet or personal computer and permitting a low level of immersion; semi-immersive consisting of huge displays or projectors that enable the patient to experience a portion of the virtual environment and the outside world at the same time while exhibiting medium level of interaction and immersion; and complete immersive involving cave automatic virtual environment (CAVE) and head-mounted display (HMD) systems and devices permitting a high level of interaction and immersion in the virtual environment while restricting the patient’s view of the actual world. The effectiveness and usefulness of VR are widely acknowledged, as it offers patients rehabilitation in a safe and customized environment while also boosting their motivation and adherence to therapy [4,16].

Empathy and understanding: therapists in the patient’s shoes

Clinical empathy is communicating with the patient, comprehending and verifying their perspective and feelings, and acting therapeutically based on that knowledge. It also includes communication with the patient and understanding their situation, feelings, and associated meanings. The expected benefits of empathy that involve ordinary behavior, decreased malpractice litigation, dutifulness, improved history taking and physical examination, moral reasoning, enhanced therapeutic relationships, patient and therapist satisfaction, and overall improved clinical outcomes are the reason clinical empathy has been integrated into the health care curriculum. Three components are included in the multifaceted idea of empathy in patient care and consist of “perspective taking”, “compassionate care”, and “standing in the patient’s shoes” [25].

Empathy is categorized into two forms, namely cognitive and affective. Cognitive empathy enables therapists to understand a patient's mental state and observe situations according to the patient’s perspectives. Whereas affective empathy demonstrates an emotional response, allowing the therapist to understand, feel, and share the emotions of the patient. This unique quality is remarkable for patient-centered intervention programs and the strengthening of therapeutic interpersonal relationships between therapists and patients. Empathy, according to research, enables therapists to offer the appropriate type of support, improve patient outcomes, and elevate patient satisfaction, all of which help to reduce errors and improve overall rehabilitation [26,27]. 

The advent of VR-based rehabilitation yielded favorable results in stimulating the understanding of behavior and empathy among therapists. VR platforms can provide users with an immersive experience of another individual or create an environment to practice and improve skills. A well-designed VR experience may include a variety of components intended to provide a believable, realistic experience in which a user may engage, relate to, and learn from the virtual surroundings. These could include immersion, in which incorporated multisensory stimuli simulate first-person perspective in real time; agency, which strengthens the illusion of control over one’s actions within the experience; and perceptual illusions, in which a person has a strong sense that they are actually in the environment. For therapeutic purposes, the integration of empathy may be enhanced through VR experiences in which the therapist either assumes the patient’s condition and perspective or operates in a virtual practice setting with virtual patients or individuals [26]. 

VR is adaptable enough to accommodate a range of circumstances, as evidenced by its capacity to encourage empathetic behavior among therapists working with patients suffering from abdominal pain, cranial nerve injury, breast cancer, and dementia [26]. A more empathetic approach to treatment is fostered by this greater understanding, which enables therapists to customize therapy to better suit patient’s physical and emotional requirements [28]. VR offers additional, possibly less evident advantages for psychotherapy. For instance, it might provide privacy and lessen embarrassment in performing treatments that would otherwise need to be given in front of others [15].

Together, the patient and the therapist may rebuild a sequence of essential stimuli that support the medical condition. Following this, a program of desensitization can be designed to expose the patient to the experience in a way that is appropriate for the demands of the disorder and the degree of severity of the condition. The patient can convey his emotions, feelings, and thoughts to the therapist in this manner, which improves mutual comprehension while also allowing the therapist to observe the patient "live" through the difficult scenarios [29]. The concept behind the simulation is to provide the patient access to all the emotions that led to the trauma but in a safe environment that is under the therapist’s observation. The possibility is offered by completely immersing oneself in the prior experience and having the ability to go gently, omitting anything for which the patient is unprepared [29]. Thus, by using VR, therapists can experience both the pros and cons of the technology, giving them useful insight into how to better implement the technology in rehabilitation. This practical experience is essential for detecting potential challenges, such as discomforts or difficulties navigating in the VR environment, and modifying the therapy as needed to optimize efficacy and patient comfort [4,29].

Innovative treatment approaches

VR-based rehabilitation allows for the application of novel innovative treatment strategies that go beyond conventional techniques. Expert VR therapists are more likely to create advanced therapy regimens that benefit from the technology’s special features [5,22]. Tasks tailored to an individual’s cognitive and physical disorders can be created in virtual environments, which is essential for reactivating the brain regions involved in motor planning, learning, and execution, as well as in maintaining patient interaction [30].

Today, VR-based telerehabilitation is an active research interest. Therapists employing VR-based telerehabilitation can prescribe therapy sessions through the Internet, which patients can readily access and carry out from the comfort of their homes. The real-time clinical measure collection and storage occurs in online databases that are accessible remotely. This means that without requiring in-person interaction or training, therapists can track their patient’s progress online and modify the therapy as needed. This represents a significant advancement over the traditional face-to-face rehabilitation paradigm and a significant cost savings for therapists as it allows them to monitor multiple patients exercising concurrently at home [31,32]. 

Another innovative approach in VR-based rehabilitation involves the incorporation of gamification components, which have been demonstrated to boost patient interaction and motivation. Patient rehabilitation can be made more effective and pleasurable by designing VR environments with game-like elements including targets, interactive challenges, and rewards [20]. Exergaming sessions made possible by gamification are simple, fascinating, interactive, and easy to comprehend and carry out [33]. The benefits of gamification in clinical practice for patients with traumatic brain injuries, lower limb amputation, cerebral palsy, and chronic low back pain have been demonstrated in recent literature [34-36]. Gamification seems to be a simple, painless, non-invasive, immersive, fascinating, and interactive method that takes into account flow, significant rewards, and the emergence of innovative solutions for patient education, evaluation, and rehabilitation. Gaming experiences additionally influence the motivation and behavior of patients [33].

The advantages of various innovative treatment approaches consist of customization to patient needs, preferences, and treatment goals; individualized therapy without demanding an intense one-on-one time commitment from the therapist; targeted improvements in physiological, motor, and/or cognitive performance; ease of transportability into the home; immediate feedback to the patient; a practical and efficient means of continuing care; and a record of quantitative performance data that can be easily accessed by the therapist [20].

Challenges in the implication of VR

The main issues brought about by the use of VR in healthcare include a reduction in face-to-face interaction. For instance, in telerehabilitation using VR to conduct rehabilitation exercises at home or elsewhere without the direct supervision of a therapist or their physical presence may result in unfavorable outcomes. These issues can be resolved by first conducting patient education through face-to-face communication at the healthcare organization and managing the treatment process under the supervision of a professional. Additionally, the financial costs of VR technology are considered a significant challenge because they necessitate expensive hardware, fast computers, effective graphics cards, high-resolution displays, precise tracking systems, and highly specialized accessories. To ensure the success of these projects, it is imperative that the therapeutic and educational programs be designed and implemented by a team of experts and that their funding should be prioritized [2].

Furthermore, a complete manual outlining where, how, and for whom VR technology in medical education and treatment is applicable is required specifically in the field of psychiatry. For this, demographic information of patients such as gender, age, personality, and history of motion sickness, as well as other distinctive cognitive, physical, functional, and psychological characteristics that are common among some clinical conditions are important to be taken into consideration and should be recorded [2,37]. Moreover, the most important non-technological component in VR use is the therapist’s and the patient’s attitude toward the technology’s acceptance. In addition, there may be ethical or legal issues arising from the recent technological advancements, resistance from some patient groups regarding using VR in treatment, and induced side effects of VR, such as cybersickness, which causes vomiting, ataxia, nausea, dizziness, and eye fatigue [2,15]. Identifying these potential challenges may help therapists to make decisions strategically on the application and advancement of these technologies in the healthcare sector.

## Conclusions

In conclusion, for both therapists and patients, the usability and user experience of VR technology in rehabilitation therapy are essential. It enhances productivity, develops empathy, and encourages innovation in treatment methods for therapists, whereas for patients it provides customized, fascinating, effective, and interactive rehabilitation experiences. As VR technology is advancing, it is expected to perform a progressively important part in therapeutic rehabilitation, emphasizing the necessity for persistent advancement and research in this field. Implementing VR technology in rehabilitation not only enhances present therapy approaches but also creates opportunities for future advancements that could completely transform patient care in the future.
